# Posterior-Chain Resistance Training Compared to General Exercise and Walking Programmes for the Treatment of Chronic Low Back Pain in the General Population: A Systematic Review and Meta-Analysis

**DOI:** 10.1186/s40798-021-00306-w

**Published:** 2021-03-08

**Authors:** Nicholas Tataryn, Vini Simas, Tailah Catterall, James Furness, Justin W. L. Keogh

**Affiliations:** 1 Department of Physiotherapy, Faculty of Health Science and Medicine, Bond University, Gold Coast, QLD Australia; 2 Water Based Research Unit, Department of Physiotherapy, Bond University, Gold Coast, Australia; 3 Faculty of Health Sciences and Medicine, Bond University, Gold Coast, Australia; 4 Sports Performance Research Centre New Zealand, AUT University, Auckland, New Zealand; 5 Cluster for Health Improvement, Faculty of Science, Health, Education and Engineering, University of the Sunshine Coast, Sippy Downs, Australia; 6 Kasturba Medical College, Mangalore, India; 7 Manipal Academy of Higher Education, Manipal, Karnataka India; 8 Bond University, Robina, QLD 4226 Australia

**Keywords:** Chronic low back pain, Resistance training, Posterior chain, Pain, Deadlift, Aerobic training

## Abstract

**Background:**

While chronic exercise training has been demonstrated to be an effective non-pharmacological treatment for chronic low back pain (CLBP), there has been a relative lack of evidence or clinical guidelines for whether a posterior chain resistance training programme provides any benefits over general exercise (GE).

**Objectives:**

To determine if chronic posterior chain resistance training (PCRT), defined as exercise programmes of ≥6 weeks duration focused on the thoracic, lumbar and hip extensor musculature, is more effective than GE in improving pain, level of disability, muscular strength and the number of adverse events in recreationally active and sedentary individuals with CLBP.

**Methods:**

Four electronic databases were systematically searched from 25 September 2019 until 30 August 2020. Using the Joanna Briggs Institute (JBI) Critical Appraisal Tools checklist for randomized controlled trials (RCTs), articles were critically appraised and compared against the inclusion/exclusion criteria. Standardized mean difference (SMD), risk difference (RD) and confidence interval (CI) were calculated using Review Manager 5.3.

**Results:**

Eight articles were included, with a total of 408 participants (203 PCRT, 205 GE). Both PCRT and GE were effective in improving a number of CLBP-related outcomes, but these effects were often significantly greater in PCRT than GE, especially with greater training durations (i.e. 12–16 weeks compared to 6–8 weeks). Specifically, when compared to GE, PCRT demonstrated a greater reduction in pain (SMD = − 0.61 (95% CI − 1.21 to 0.00), *p = 0.05*; *I*^2^ = 74%) and level of disability (SMD = − 0.53 (95% CI − 0.97 to − 0.09), *p = 0.02*; *I*^2^ = 52%), as well as a greater increase in muscle strength (SMD = 0.67 (95% CI 0.21 to 1.13), *p = 0.004*; *I*^2^ = 0%). No differences in the number of adverse events were reported between PCRT and GE (RD = − 0.02 (95% CI − 0.10 to 0.05), *p = 0.57*; *I*^2^ = 72%).

**Conclusion:**

Results of the meta-analysis indicated that 12–16 weeks of PCRT had a statistically significantly greater effect than GE on pain, level of disability and muscular strength, with no significant difference in the number of adverse events for recreationally active and sedentary patients with CLBP. Clinicians should strongly consider utilizing PCRT interventions for 12–16 weeks with patients with CLBP to maximize their improvements in pain, disability and muscle strength. Future research should focus on comparing the efficacy and adverse events associated with specific PCRT exercise training and movement patterns (i.e. deadlift, hip lift) in treating this population.

**Trial registration:**

PROSPERO CRD42020155700.

## Key Points

- Posterior chain resistance training is more effective in reducing pain and disability and improving muscle strength in patients with chronic low back pain than general exercise.
- Posterior chain resistance training does not have significantly more adverse events than general exercise in patients with chronic low back pain.
- Clinicians should strongly consider the prescription of 12–16 weeks of posterior chain resistance training to maximize outcomes in their patients with chronic low back pain.

## Introduction

Chronic low back pain (CLBP) is a complex multifaceted condition and is one of the most prevalent medical disorders in today’s societies, being the leading cause of disability and amounting to an alarming worldwide economic cost, with more than $100 billion annually in the USA alone [1–3]. CLBP is classified as back pain localized above the gluteal fold, and below the costal margin, that persists for a minimum duration of 12 weeks [4]. CLBP accounts for ~ 80% of the direct cost of low back pain, with clinicians unable to make a specific diagnosis in up to 90% of patients, therefore classifying patients as having nonspecific CLBP [5]. Although acknowledged as a multifactorial condition, it has also been suggested that deconditioning of the posterior chain muscles, i.e. those found in the thoracic, lumbar and posterior hip region [6], is a contributing risk factor for low back pain [7]. Maintaining sufficient muscular strength and endurance as well as adequate levels of motor control of the posterior chain musculature that allow the performance of a variety of occupational, sport and recreational activities with minimum risk of injury should be considered an important goal of this rehabilitation [6].

CLBP affects a widespread population of people, regardless of their current levels of sedentary behaviour or physical activity [8]. In particular, the disability associated with CLBP may result in substantial losses in quality of life and posterior chain function, which may be related to the increase in pain and the fear-avoidance cycle that recurrently foreshadows the onset of pain [8, 9]. These declines in function and quality of life appear consistent with the view that a variety of neurophysiological, social and psychological factors negatively influence individuals with CLBP [10].

Exercise therapy has been demonstrated to be effective in decreasing pain compared with non-exercise-based treatments in adults [11], with such improvements thought to reflect some combination of the exercise-related physical and psychological adaptations. Exercise therapy is the most widely used non-pharmacological treatment, comparable to other conservative treatments for improving disability and pain intensity in CLBP [8, 12, 13]. Two recent systematic reviews reported that exercise training is an effective treatment for reducing pain in patients with CLBP; however, they both highlighted that it is still unclear exactly what forms of exercises are most effective or which mechanisms may most likely explain such improvements [8, 13, 14]. As a result, current clinical updates regarding exercise prescription for chronic musculoskeletal pain, including CLBP, recommend graded and individualized resistance or aerobic exercise at low to moderate intensities, performed 2 days per week for 6 weeks to improve pain intensity and level of disability [8].

Such clinical guidelines still leave many questions unanswered. For example, if a clinician was to prescribe resistance training for individuals with CLBP, there is currently no consensus on what exercises should be included or excluded in the exercise programme in order to target particular movements, joints and/or muscles relevant to the individual’s CLBP.

As an example, a common method used by physiotherapists in diagnosing and treating musculoskeletal disorders is to assess and treat the major joints above and below the affected area. In the case of CLBP, these joints would be the thoracic spine and the hips. However, there are currently no clinical guidelines or recommendations for how resistance training should target these additional regions in a graded exercise programme for patients with CLBP.

Another issue with current guidelines is that much of the literature equating resistance and aerobic exercise for the treatment of CLBP have been provided with respect to reducing self-reported pain intensity, typically assessed on a 10-point pain scale. While this focus on pain is understandable, a greater understanding of how these exercise modes may influence other treatment outcome measures including level of disability, muscular strength and potential adverse events associated with the exercise therapy may be useful for clinicians who work with patients with CLBP [8].

Therefore, the aim of this study was to conduct a systematic review and meta-analysis to evaluate the effectiveness of specific kinds of exercise training, specifically posterior chain resistance training (PCRT) compared to either aerobic exercise or general exercise, in sedentary or recreationally active populations with CLBP. The level of pain, level of disability, muscular strength and adverse events were selected to provide a more comprehensive description of the potential benefits and risks of different exercise modalities currently recommended for people with CLBP.

## Methods

### Protocol and Registration

This systematic review and meta-analysis adhered to the Preferred Reporting Items of Systematic Reviews and Meta-Analyses (PRISMA) guidelines [15] and has been registered with PROSPERO (CRD42020155700).

### Inclusion Criteria

Studies considered for inclusion were English-language randomized controlled trials (RCTs). In order to be eligible for the study, the sample described in each study had to include participants with a mean age of 16–70 years old. Further, all individuals had to have a diagnosis of, or were presenting with CLBP (localized above the inferior gluteal folds and below the costal margin, with or without leg pain) for a minimum of 12 weeks [4], which did not have a known aetiology [11]. Studies were required to include at least one of the outcome measures of interest: pain, disability, muscle strength or adverse events. Both the experimental and comparator interventions must be entirely land-based as well, meaning all aqua therapy exercise interventions were excluded from this review.

For the purpose of this review, the term “posterior chain resistance training” was defined as exercises and movements that targeted muscles located in the thoracic, lumbar and posterior hip regions that were agonists for hip extension, lumbar and/or thoracic extension, shoulder extension, scapular downward rotation, scapular elevation and scapular retraction. This definition sought to quantify muscle activity in a range of posterior chain resistance training exercises [6].

As comparator groups, we considered interventions of “general exercise” (GE), being any walking interventions or resistance training regime that did not meet the criteria of the majority “posterior-chain exercise” intervention, with these interventions required to be graded or progressive in nature. The general exercise intervention groups were included if administered ≥ 1 session/week for a duration of ≥ 30–45 min for ≥ 6 weeks. The walking intervention groups were included if administered ≥ 1 sessions/week of ≥ 20–30 min duration for ≥ 6 weeks. These respective standards are objectively lower than current standards for the general population which are 30 min a day for 5 days a week of moderate intensity physical activity [16]. The authors recognize that those with CLBP may need to start substantially lower than the daily recommendations for physical activity of the general population due to deconditioning, pain and fear avoidance that have resulted from long periods of inactivity and/or disability.

### Exclusion Criteria

Excluded studies included those in which the participants had CLBP due to or associated as a symptom of a known spinal condition. Such spinal conditions may be characterized as structural (e.g. spondylosis, spondylolisthesis, herniated disc, scoliosis), non-mechanical (e.g. ankylosing spondylitis, neoplasia, infection, inflammation, osteoporosis, radicular syndrome, radiculopathy, cauda equina syndrome, cerebral palsy) or referred pain (aortic aneurysm, diseases of the pelvic organs, gastrointestinal disease or renal disease) in nature [17, 18]. Studies inclusive of participants undergoing rehabilitation post-surgery for a low back condition/injury or pre-surgery with an upcoming planned surgery were excluded. Studies including competitive/elite level athletes were excluded. Any studies with water-based interventions (i.e. aquatic therapy, aquatic aerobics) or use of manual therapy and/or electronic modalities were also excluded.

### Search Strategy

Four electronic databases were searched from 25 September 2019 up until 30 August 2020: PubMed, CINAHL, SPORTDiscus and Embase. The search strategy included key terms related to posterior chain resistance training, exercise, low back pain and quality of life. Table 1 outlines the search terms applied to individual databases. Two independent reviewers (NT and TC) screened the titles and abstracts of the studies against the inclusion and exclusion criteria. Reference lists of included papers were reviewed for potential inclusions. The reviewers further assessed full-text papers for inclusion; a third reviewer (JK) moderated any discrepancies.

**Table 1:** Applied search terms during systematic review

Databases	Search terms
PubMed, CINAHL, SPORTDiscus, Embase	(“Posterior chain ‘s exercise” OR deadlift OR “hip lift” OR “back extensions” OR “back training” OR “back exercise” OR “resistance training” OR “resistance exercise” OR “physical exercise” OR “physical activity”) AND (“Low back pain” OR “chronic non-specific low back pain” OR “back pain” OR “chronic low back pain”) AND (“Quality of life” OR “pain” OR “function” OR “activities of daily living” OR “ADL” OR “strength” OR “movement”) NOT (“hydrotherapy” OR “water exercise” OR “swimming”)

### Data Extraction

Upon finalization of the literature search, all results were exported to an external citation management software (EndNote X92, Clarivate Analytics), whereby the separate database searches were combined, and all duplicate records removed. Two independent reviewers (NT and TC) then screened the titles and abstracts to exclude studies not eligible for consideration. For those studies deemed potentially eligible, a full text was obtained, and the eligibility criteria was applied to determine inclusion in the systematic review. Study design, relevant publication information (e.g. title, year, author, journal), number of participants, participant characteristics (e.g. age, sex, level of CLBP), considered interventions and comparator groups, inclusive of length all of intervention and outcome measures (i.e. any measures of pain intensity, level of disability, adverse events and muscular strength) were extracted by two independent reviewers (NT and TC). Data for each of the outcome measures were obtained where possible at baseline, post-intervention and change during intervention using measures of centrality and dispersion, such as the mean and standard deviation (SD). If a *p* value or SD could not be retrieved, the available data were converted to a CI.

### Study Quality and Critical Appraisal

Study quality was assessed using the Joanna Briggs Institute (JBI) Critical Appraisal tools checklist for RCTs [19]. Two reviewers (NT and TC) independently scored each of the RCT studies in the 13 relevant categories, obtaining a final decision to include the study. The reviewers discussed any discrepancies and consulted a third reviewer (JK) to resolve any differences. Cohen’s kappa analysis was then run for reviewer 1 and 2 before obtaining the overall kappa value. Descriptors of overall quality of the studies included were based on Kennelly et al. [20]. Due to modification required in previous research [21], scores were converted to percentages to enable the proposed quality rating. The following quality rating ranges were applied to the converted raw score percentage: < 45.4% were considered “poor” quality, between 45.4 and 61.0% were considered “fair” and over 61% were considered of good methodological quality.

### Data Analysis and Publication Bias

Where possible, quantitative data were entered into Review Manager (RevMan) software, version 5.3 (The Nordic Cochrane Centre, The Cochrane Collaboration, Copenhagen, 2014), to perform statistical calculations. Statistical analysis was completed where effect sizes and their 95% CI were reported, with Hedges’ g used to account for bias in effect sizes. To standardize the results of the studies to a uniform scale, pain, disability and muscular strength data were expressed using the standardized mean difference (SMD) and 95% CI. SMD was calculated through SPSS using the difference in mean outcome between groups, divided by the standard deviation of outcome among participants, which is consistent with the standards of the Cochrane Back and Neck Group [22]. For adverse events, the risk difference (RD) and 95% CI was used as the measure of effect size. To show the overall magnitude of effect for each subgroup, SMD > 0.8 represented a large clinical effect, 0.5–0.79 a moderate effect and 0.2–0.49 a weak effect [23]. A modification to this approach used in other studies [23–25] was added where 0.00–0.19 was expressed as a “trivial” finding.

Heterogeneity was assessed statistically using the *I*^2^ test which is a common approach for meta-analysis [26]. Interpretation of heterogeneity was based on an adapted version of the JBI reviewer’s manual [19], as has previously been done in systematic reviews [8]. Using the guidelines from the Cochrane Handbook for Systematic Reviews and Interventions [27], *I*^2^ values between 0 and 40% might not be important, 30–60% may represent moderate heterogeneity, 50–90% may represent substantial heterogeneity and > 75–100% represents considerable heterogeneity. Where heterogeneity was moderate or higher, a chi-square sensitivity analysis was conducted. For each outcome measure used, the effect was calculated based on the difference between interventions from baseline to follow-up and variance was calculated based on the SD. Where SD was not available, either *p* values or CI were used to provide an estimate of variance. When *p* values for a significant difference were not available, a conservative approach was used, where *p < 0.05* was imputed. Similarly, for results where *p* values were not statistically significant, a value of *p < 0.1* was used. Utilizing the criteria outlined in Table 2, set out by Furlan et al. [22], level of evidence was synthesized for each of the 4 outcome measures with subgroup analysis. Test for funnel plot asymmetry was used to investigate the risk of publication bias.

**Table 2:** Levels of evidence approach [22]

Level of evidence	Criteria
Strong	Consistent^a^ findings from 3 or more high-quality studies
Moderate	Consistent^a^ findings from at least 1 high-quality and 1 low-quality study
Limited	Consistent^a^ findings in > 1 low-quality study or only one study available
Conflicting	Inconsistent evidence in multiple studies irrespective of study quality
No evidence	No studies found

^a^Consistent was interpreted as > 60% of studies trending from the line of no effect in either direction

## Results

### Search Strategy Results

**Fig. 1:**
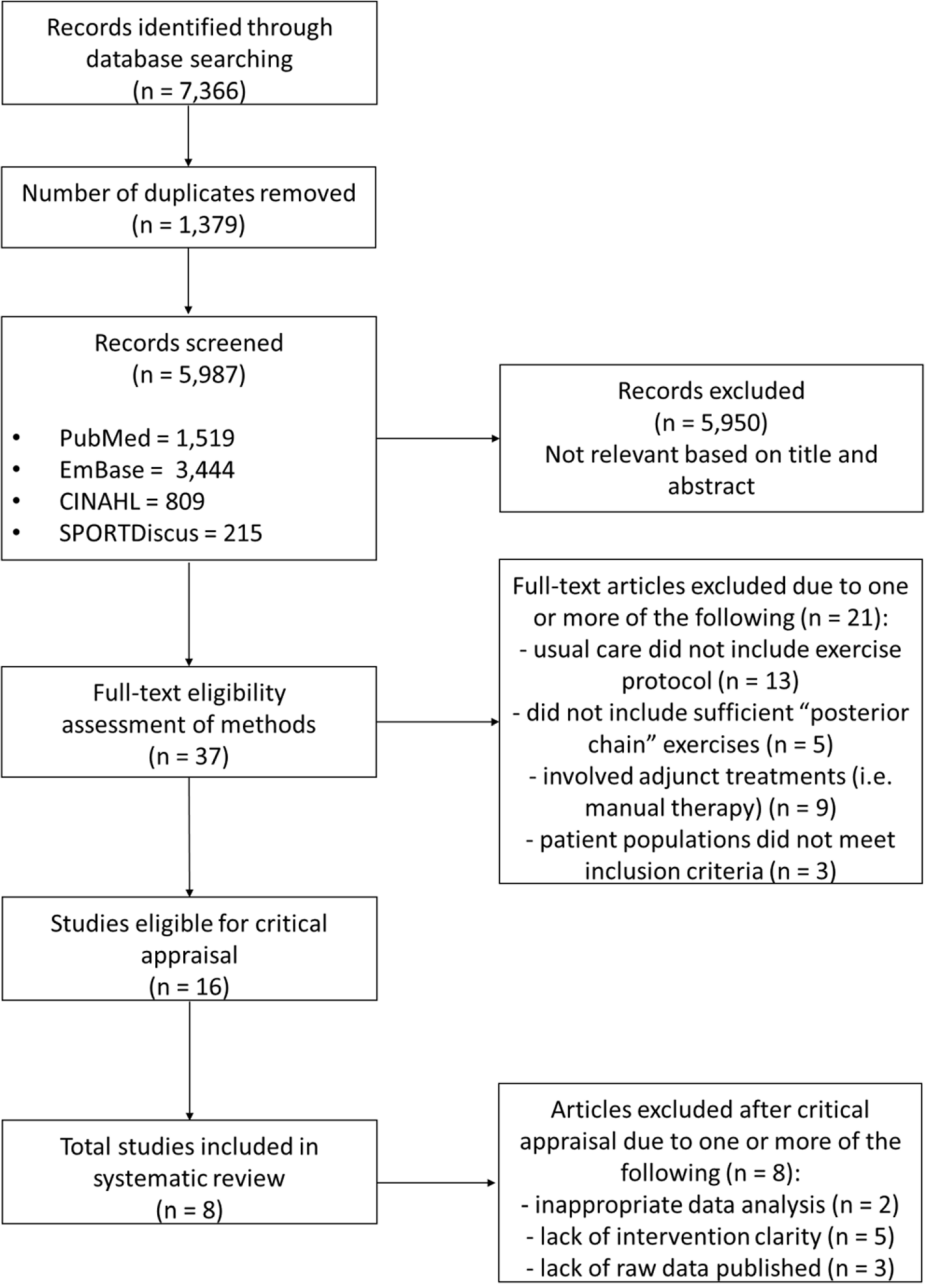
PRISMA flow diagram

A summary of the search results is provided in the PRISMA flow diagram presented in Fig. 1. The total number of results obtained from the databases was 7366. Following the removal of 1379 duplicates, 5987 articles were screened. Of those screened, 5950 were excluded for not meeting the inclusion criteria or falling within the exclusion criteria, leaving 37 articles for full-text screening. Sixteen articles were deemed appropriate for critical appraisal, with this further narrowed down to 8 articles, as 8 were excluded due to either inappropriate analysis, lack of results or a lack of sufficient detail in regard to the exercise prescription.

### Quality of Assessment Results

The two independent reviewers (NT and TC) reached an “almost perfect” level of agreement on their critical appraisal of the included studies according to results of the kappa analysis (*k* = 0.896) [28, 29]. Seven studies were rated as “good” [14, 30–35] and one was rated as “fair” [36], with a summary of this provided in Table 3. As a result, all studies were included in the review.

**Table 3:** Risk of bias assessment of RCTs

Study	Risk of bias assessment of the studies													
	1	2	3	4	5	6	7	8	9	10	11	12	13	Overall risk of bias rating (%)
Aasa et al. 2015 [30]	+	+	–	n/a	n/a	+	+	+	+	+	+	+	+	77
Atalay et al. 2017 [36]	+	–	–	n/a	n/a	–	+	+	+	+	+	+	+	62
Cai et al. 2015 [31]	+	+	+	n/a	n/a	+	+	+	+	+	+	+	+	85
Cortell-Tormo et al. 2018 [14]	+	+	+	n/a	n/a	–	+	+	+	+	+	+	+	77
Hurley et al. 2015 [32]	+	+	–	n/a	n/a	+	+	+	+	+	+	+	+	77
Jackson et al. 2010 [33]	–	+	+	n/a	n/a	–	+	+	+	+	+	+	+	69
Kell et al. 2009 [34]	–	+	+	n/a	n/a	+	+	+	+	+	+	+	+	77
Suh et al. 2019 [35]	+	–	–	n/a	n/a	+	+	+	+	+	+	+	+	69
Number of studies with a “yes” response	6	6	4	0	0	5	8	8	8	8	8	8	8	

All studies included in this table were completed under the JBI Critical Appraisal Tool for RCTs. 1. Was true randomisation used for assigning participants to treatment groups? 2. Was allocation to treatment groups concealed? 3. Were treatment groups similar at the baseline? 4. Were participants blind to treatment assignment? 5. Were those delivering treatment blind to treatment assignment? 6. Were outcomes assessors blind to treatment assignment? 7. Were treatment groups treated identically other than the intervention of interest? 8. Was follow-up complete and if not, were differences between groups in terms of their follow-up adequately described and analysed? 9. Were participants analysed in the groups to which they were randomized? 10. Were outcomes measured in the same way for treatment groups? 11. Were outcomes measured in a reliable way? 12. Was appropriate statistical analysis used? 13. Was the trial design appropriate, and any deviation from the standard RCT design accounted for in the conduct and analysis of the trial?

It is worth noting that Question 4 regarding blinding of the participants and Question 5 regarding blinding of the treatment administrators were deemed “not applicable” for all studies completed using the JBI Critical Appraisal Tool for RCTs. Due to the nature of participants actively taking part in their treatment and treatment administrators knowingly teaching/coaching the interventions, it is impossible to blind either party in these studies. The questions were still counted in the final score as a “no” response though.

### Characteristics of Included Studies

Studies included were of diverse origins: Sweden [30], Turkey [36], Singapore [31], Spain [14], Ireland [32], Canada [33, 34] and Korea [35]. The populations outlined gave specifics on sex of the participants: males alone [33, 36], females alone [37] or mixed sexes [30–32, 34–36]. Three studies identified their group activity levels specifically: recreational runners [31], recreationally active [33] or sedentary [36].

In terms of GE interventions, seven used resistance and/or walking programmes that did not fit the definition of a PCRT intervention [30–36], and one used activities of daily living (ADLs) [14]. The study utilizing ADL was included as the authors stated that the control group was only told not to complete exercises like the ones performed by the PCRT group. The length of interventions varied from 6 to 16 weeks. They were subsequently categorized into 2 groups based on training duration: 6–8 weeks [30, 31, 35, 36] or 12–16 weeks [14, 32–34]. Specific aspects of each study can be found in Table 4.

**Table 4:** Characteristics of included studies

Study	Number of participants	Age	Sex(es)	Structure of PCRT session	Number of sessions/week	Sets × reps/rest of each exercise	Type of loading	Exercises included in PCRT intervention
**6–8 weeks**								
Aasa et al. 2015 [30]	70	25–60 years	M + F	Small group (5 people)	1–2×/week	5 + × 1–10/5 min (based on total load)	BB	Deadlift
Atalay et al. 2017 [36]	20	25 years (0.8 SEM)	M	Circuit-based, changing after each set	3×/week	2 × 6–12 reps or 5 s holds/30 min between circuits	BW, DB	Bridge, weighted neck flexion/extension, lateral side raise, upright row, chest-supported DB row, abdominal crunch, 4-point kneeling w/ leg extension, supine back extension
Cai et al. 2015 [31]^a^	84	21–45 years	M + F	In-clinic and home exercises	Clinic: 2×/week Home: 5×/week	Clinic: 3 × 10/2 min Home: 3 × 10/2 min	BW, DB, Machine	Supervised: Machine-based hip abduction, hip extension, leg press Home Exercise: Single-leg squat, wall sit
Suh et al. 2019 [35]^a^	48	54.81 years (± 14.66)	M + F	Home-based	5×/week	5 × 30 s holds (rest not mentioned)	BW, DB, varying level of instability	Bridge, abdominal crunch, dead bug, side plank, prone superman, bird dog, plank
**12–16 weeks**								
Cortell-Tormo et al. 2018 [14]	19	20–55 years	F	Small group (3–4 people), circuit-based	2×/week	Stage 1: 1–2 × 20/0 s Stage 2: 2–3 × 15/30 s Stage 3: 3 × 12/30 s	BW, DB, Cable; based on RPE of each participant	Isometric: 4-pt kneeling/lying, lying w/ leg movement, sitting, standing Movements: Squat, lunge, SL deadlift, stand row, stand push, seated row, pull squat, abdominal crunch, back extension, side plank, bridge
Hurley et al. 2015 [32]	246	18–65 years	M + F	Group-based circuit training	1×/week	1× max reps in 60 s at 70% RPE (Borg)	BW, DB, progression to unilateral	Bridge (SL or DL), squats (SL or DL), lateral stepping, push-ups, side-lying hip abduction, prone leg extension, abdominal crunch, lateral raises, diagonal trunk curl, high-knees on the spot
Jackson et al. 2011 [33]^a^ [Middle-aged]	45	52 years (± 2.7)	M	Gym-based, unsupervised	4×/week	3–6 × 10–12/1–2 min “Core” = 30 reps	BW, DB, BB, Cable	Leg press, leg extension, leg curl, bench press, incline bench press, lat pulldown, low cable row, shoulder press, arm curl, triceps pushdown, ab crunches, Swiss ball crunch, prone superman
Jackson et al. 2011 [33]^a^ [Old-aged]	45	63 years (± 3.1)	M	Gym-based, unsupervised	4×/week	3–6 × 10–12/1–2 min “Core” = 30 reps Progressive RT	BW, DB, BB, Cable	Leg press, leg extension, leg curl, bench press, incline bench press, lat pulldown, low cable row, shoulder press, arm curl, triceps pushdown, ab crunches, Swiss ball crunch, prone superman
Kell et al. 2009 [34]	27	40.1 years (± 8.7)	M + F	Gym-based, unsupervised	4×/week	2–3 × 8–15/1–3 min “Core” = 30 reps Progressive RT	BW, DB, BB, Cable	Leg press, leg extension, leg curl, bench press, incline bench press, lat pulldown, low cable row, shoulder press, arm curl, triceps pushdown, ab crunches, swiss ball crunch, prone superman

*PCRT* Posterior Chain Resistance Training; *SEM* standard error mean; *M* male; *F* female; *BB* barbell; *BW* bodyweight; *DB* dumbbell; *10RM* 10 repetition max; *RPE* rating of perceived exertion; *RT* resistance training

^a^Participants were noted to be recreationally active

### Outcomes

#### Pain

**Fig. 2:**
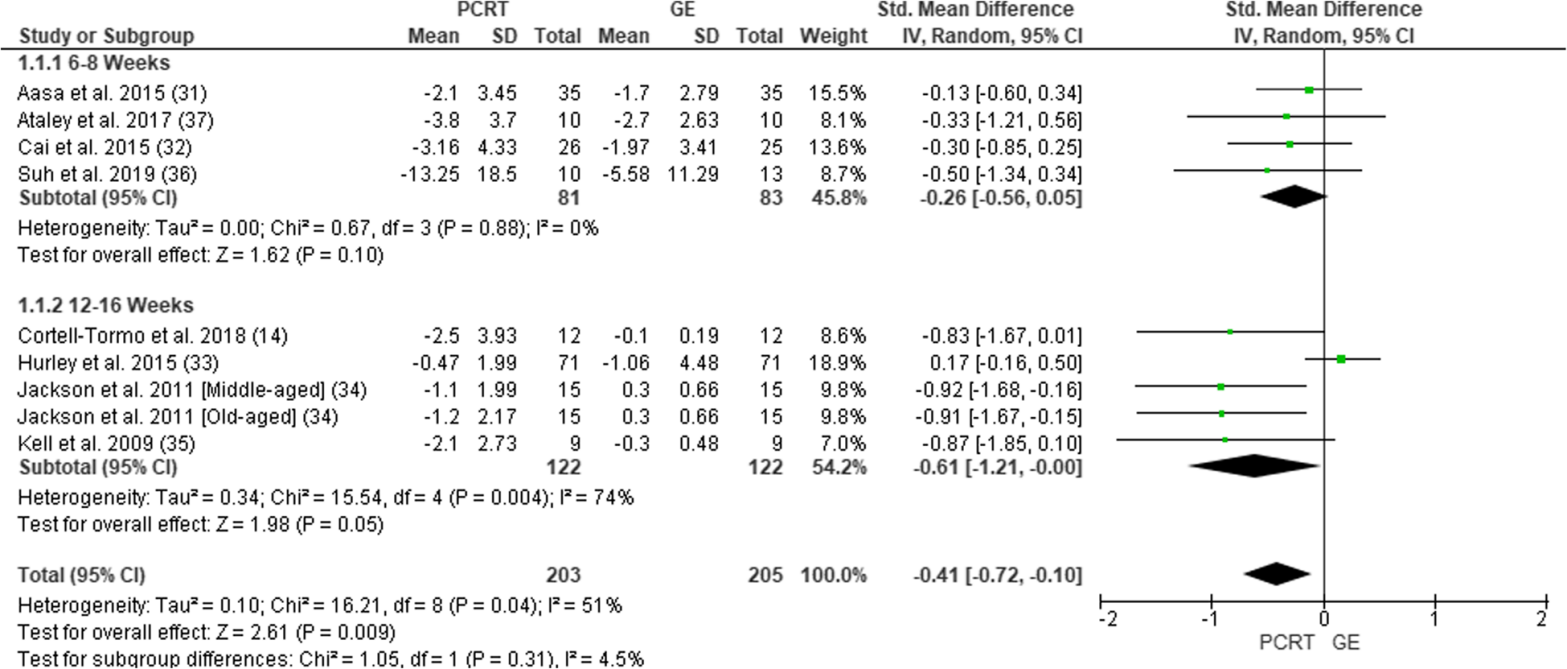
Forest plot of comparison between GE vs PCRT articles for changes in pain. SD, standard deviation; IV, inverse variable; CI, confidence intervals; PCRT, posterior chain resistance training; GE, general exercise

All eight of the included studies measured pain intensity (Fig. 2). Six studies used a visual analogue scale [38] and two used a Numeric Pain Rating Scale [39]. In total, pain intensity was assessed in 393 participants (203 PCRT; 190 GE). SMDs were analysed for the purpose of giving an overall magnitude of effect and level of evidence. The overall SMD revealed a significant change in favour of PCRT vs GE (SMD = − 0.41 (95% CI − 0.72 to 0.10), *p =* 0.009, *I*^2^ = 51%*).* Subgroup analysis revealed significant improvements with PCRT over GE in the four studies utilizing 12–16 weeks of exercise (SMD = − 0.61 (95% CI − 1.21 to 0.00), *p =* 0.05; *I*^2^ = 74%), whereas there was no statistical difference when comparing PCRT to GE over 6–8 weeks (SMD = − 0.26 (95% CI 0.56 to 0.05), *p =* 0.10; *I*^2^ = 0%).

Overall, there was moderate level of evidence suggesting a small effect size difference in supporting the use of PCRT over GE in the treatment of CLBP when measuring pain at 6–8 weeks (Table 5). When measuring pain at 12–16 weeks, there was a strong level of evidence suggesting a moderate effect size difference in favour of PCRT over GE.

**Table 5:** Best level of evidence synthesis PCRT vs GE

Study	Study quality^**a**^	Effect statistic (95% CI)	Descriptor of magnitude	Level of evidence ^**b**^
**Pain**				
**VAS and NPRS**				
**6–8 weeks**				
Aasa et al. 2015 [30]	Good	− 0.13 [− 0.60, 0.34]	Trivial	Moderate
Atalay et al. 2017 [36]	Fair	− 0.33 [− 1.21, 0.56]	Small	
Cai et al. 2015 [31]	Good	− 0.30 [− 0.85, 0.25]	Small	
Suh et al. 2019 [35]	Good	− 0.50 [− 1.34, 0.34]	Moderate	
**Overall 6–8 weeks**		− 0.26 [− 0.56, 0.05]	Small	
**12–16 weeks**				
Cortell-Tormo et al. 2018 [14]	Good	− 0.83 [− 1.67, 0.01]	Large	Strong
Hurley et al. 2015 [32]	Good	0.17 [− 0.16, 0.50]	Small	
Jackson et al. 2011 [MA] [33]	Good	− 0.92 [− 1.68, − 0.16]	Large	
Jackson et al. 2011 [OA] [33]	Good	− 0.91 [− 1.67, − 0.15]	Large	
Kell et al. 2009 [34]	Good	− 0.87 [− 1.85, 0.10]	Large	
**Overall 12–16 weeks**		− 0.61 [− 1.21, 0.00]	Moderate	
**Overall pain**		− 0.41 [− 0.72, − 0.10]	Small	Strong
**Disability**				
**ODI and PSFS**				
**6–8 weeks**				
Aasa et al. 2015 [30]	Good	− 0.38 [− 0.85, 0.09]	Small	Moderate
Atalay et al. 2017 [36]	Fair	− 0.22 [− 1.10, 0.66]	Small	
Cai et al. 2015 [31]	Good	0.15 [− 0.40, 0.70]	Trivial	
Suh et al. 2019 [35]	Good	− 0.05 [− 0.88, 0.77]	Trivial	
**Overall 6–8 weeks**		− 0.15 [− 0.46, 0.16]	Trivial	
**12–16 weeks**				
Cortell-Tormo et al. 2018 [14]	Good	− 0.91 [− 1.76, − 0.06]	Large	Strong
Hurley et al. 2015 [32]	Good	− 0.02 [− 0.35, 0.31]	Trivial	
Jackson et al. 2011 [MA] [33]	Good	− 0.74 [− 1.49, 0.00]	Moderate	
Jackson et al. 2011 [OA] [33]	Good	− 0.75 [− 1.49, − 0.00]	Moderate	
Kell et al. 2009 [34]	Good	− 0.75 [− 1.72, 0.21]	Moderate	
**Overall 12–16 weeks**		− 0.53 [− 0.97, − 0.09]	Moderate	
**Overall LOD**		− 0.31 [− 0.56, − 0.06]	Small	Strong
**Strength**				
**6–8 weeks**				
Aasa et al. 2015 [30]	Good	0.19 [− 0.28, 0.66]	Trivial	Conflicting
Atalay et al. 2017 [36]	Fair	0.41 [− 0.48, 1.30]	Small	
Cai et al. 2015 [31]	Good	0.51 [− 0.04, 1.07]	Moderate	
**Overall 6–8 weeks**		0.34 [0.00, 0.67]	Small	
**12–16 weeks**				
Jackson et al. 2011 [MA] [33]	Good	0.67 [− 0.07, 1.41]	Moderate	Strong
Jackson et al. 2011 [OA] [33]	Good	0.67 [− 0.07, 1.40]	Moderate	
Kell et al. 2009 [34]	Good	0.68 [− 0.28, 1.64]	Moderate	
**Overall 12–16 weeks**		0.67 [0.21, 1.13]	Moderate	
**Overall strength**		0.45 [0.18, 0.72]	Small	Strong

*PCRT* posterior chain resistance training; *GE* general exercise; *CI* confidence intervals; *VAS* visual analogue scale; *NPRS* Numerical Pain Rating Scale; *ODI* Oswestry Disability Index; *PSFS* Patient-Specific Functional Scale

^a^Study quality determined from quality assessment using Furlan et al. [22]

^b^Level of evidence approach criteria is shown in Table 2. Descriptors – intervention favoured

#### Disability

**Fig. 3:**
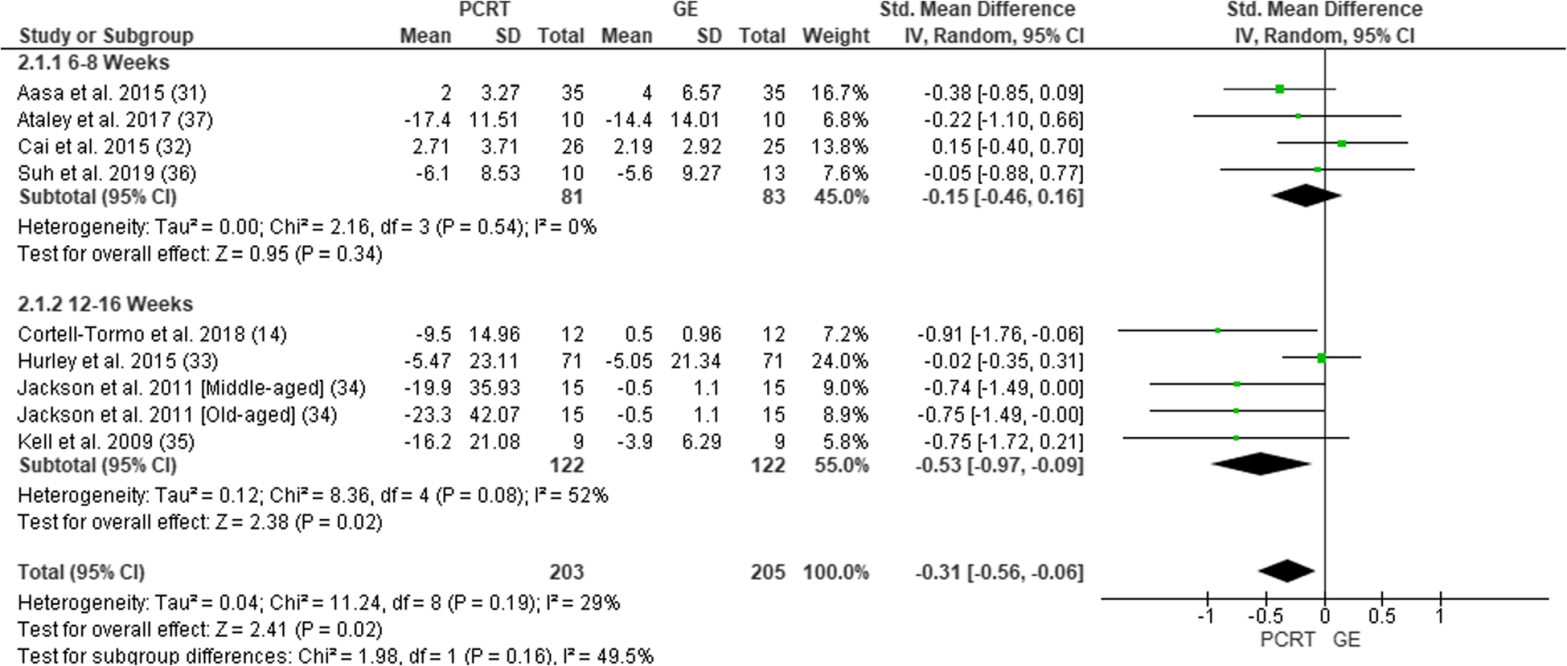
Forest plot of comparison between GE vs PCRT articles for changes in level of disability. SD, standard deviation; IV, inverse variable; CI, confidence intervals; PCRT, posterior chain resistance training; GE, general exercise

All eight of the studies measured level of disability (Fig. 3). Six studies used an Oswestry Disability Index [40] and two used a Patient-Specific Functional Scale [41]. In total, level of disability was assessed in 408 participants (203 PCRT; 205 GE). Pooled analysis of all interventions demonstrated statistically significant improvements in level of disability in the PCRT compared to the GE groups (SMD = 0.31 (95% CI − 0.56 to − 0.06), *p* = 0.02, *I*^2^ = 29%*).* Subgroup analysis revealed that PCRT resulted in significantly greater improvements in the level of disability compared to GE when the training interventions were 12–16 weeks duration (SMD = − 0.53 (95% CI − 0.97 to − 0.09), *p* = 0.02; *I*^2^ = 52%) when compared to 6–8 weeks duration (SMD = − 0.15 (95% CI − 0.46 to 0.16), *p =* 0.54; *I*^2^ = 0%).

There was moderate level of evidence suggesting a trivial effect to support the use of PCRT over GE in the treatment of CLBP when measuring level of disability at 6–8 weeks (Table 5). When measuring level of disability at 12–16 weeks, there was a strong level of evidence suggesting a moderate effect size difference supporting the use of PCRT over GE.

#### Strength

**Fig. 4:**
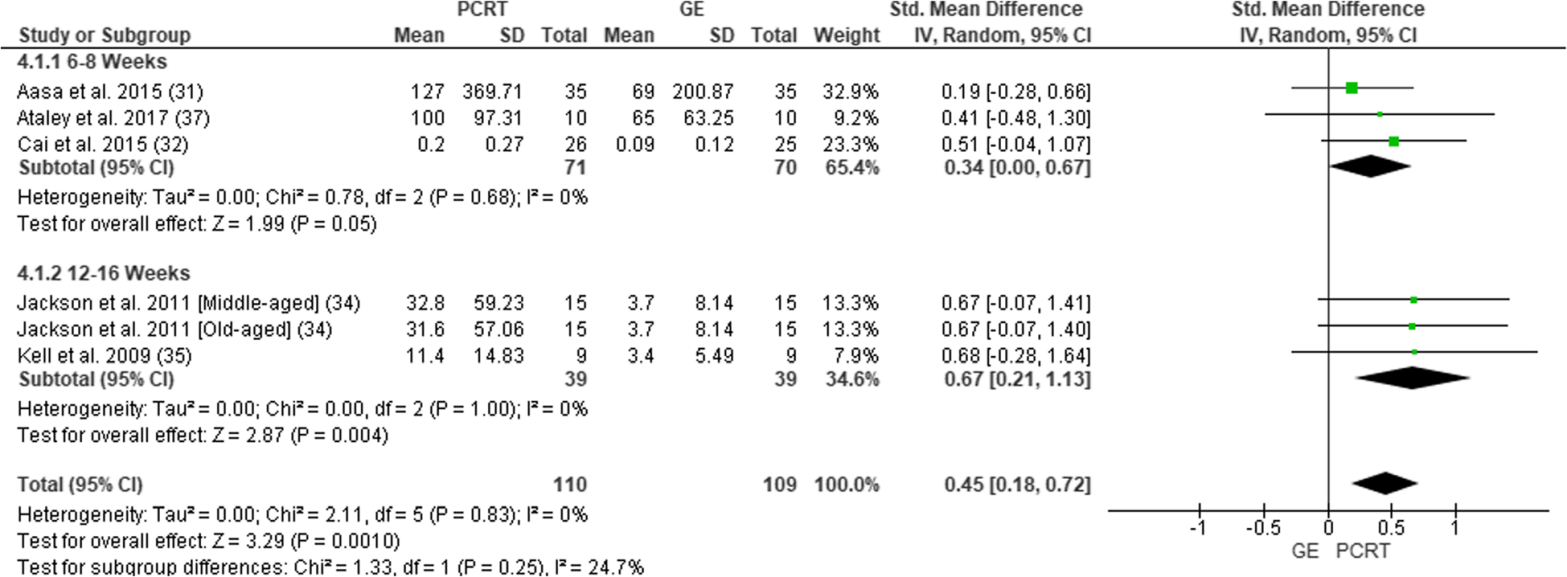
Forest plot of comparison between GE vs PCRT articles for changes in strength. SD, standard deviation; IV, inverse variable; CI, confidence intervals; PCRT, posterior chain resistance training; GE, general exercise

Five of the eight studies measured some muscular strength outcomes (Fig. 4). Measures of strength included the following: isometric lift capacity [30], isometric lumbar extension [36], knee extension torque via isokinetic dynamometer [31] and leg press for a 10-RM [33] and 5-RM [34]. In total, change in muscular strength was assessed in 219 participants (110 PCRT; 109 GE). Pooled analysis of all interventions demonstrated a statistically significant improvement in muscular strength in the PCRT groups when compared to the GE groups (SMD = 0.45 (95% CI 0.18 to 0.72), *p =* 0.001, *I*^2^ = 0%). Subgroup analysis revealed that these significant benefits of PCRT compared to GE existed for studies of 6–8 weeks duration (SMD = 0.34 (95% CI 0.00 to 0.67), *p* = 0.05; *I*^2^ = 0%) as well as 12–16 weeks (SMD = 0.67 (95% CI 0.21 to 1.13), *p =* 0.004; *I*^2^ = 0%).

When measuring muscular strength, there was conflicting evidence suggesting a small effect size difference favouring PCRT over GE over a 6–8-week period (Table 5). However, when measuring muscular strength changes over a 12–16-week period, there was a strong level of evidence suggesting a moderate effect size difference in favour of PCRT over GE.

#### Adverse Events

**Fig. 5:**
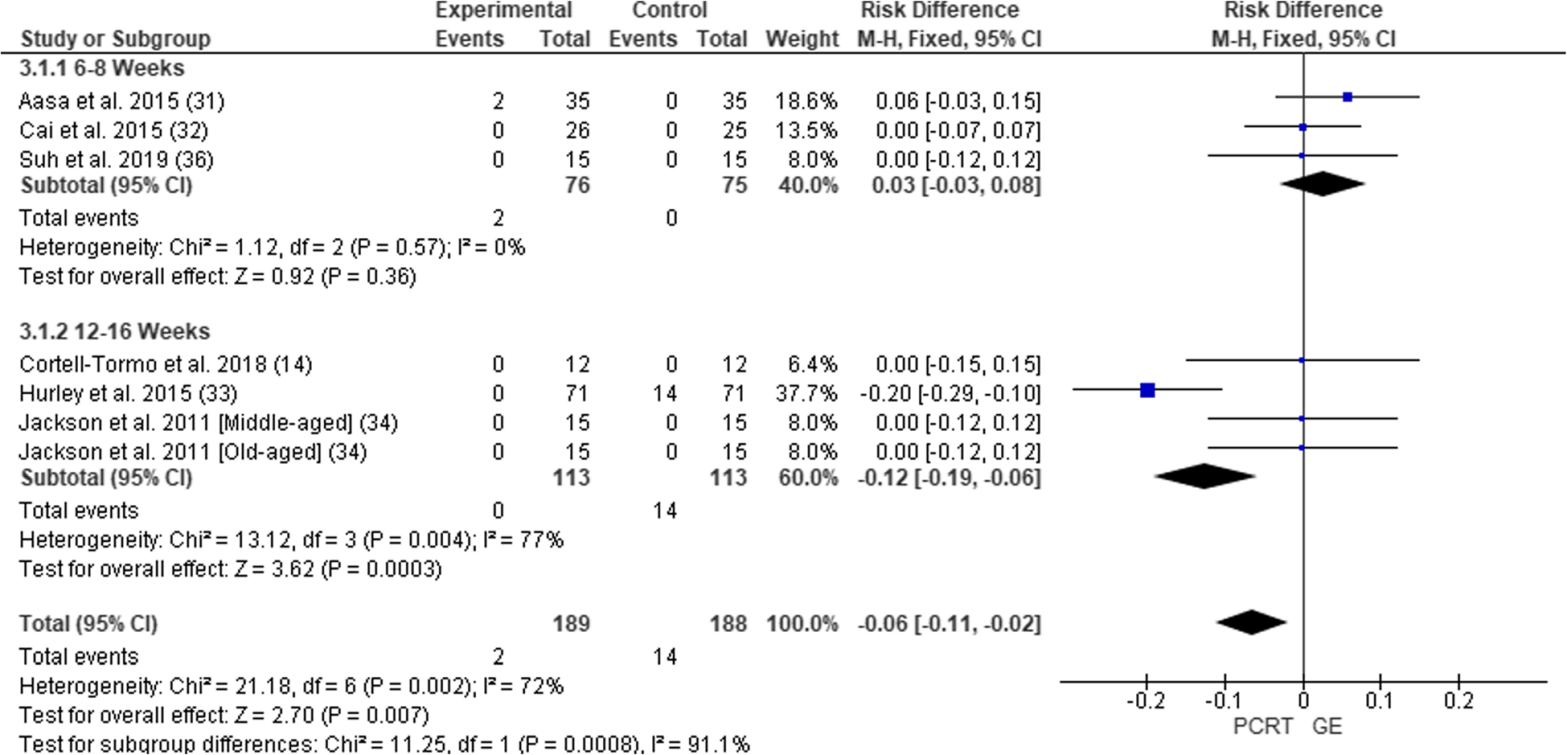
Forest plot of comparison between GE vs PCRT articles for changes in adverse events. SD, standard deviation; IV, inverse variable; CI, confidence intervals; PCRT, posterior chain resistance training; GE, general exercise

Six of the eight studies reported data for adverse events (Fig. 5). In total, adverse events were assessed in 377 participants (189 PCRT; 188 GE). Pooled analysis of all interventions indicated no significant difference in the risk of adverse events for PCRT groups compared to GE groups (RD = − 0.02 (95% CI − 0.10 to 0.05), *p* = 0.57, *I*^2^ = 72%*).* The article by Jackson et al. [33] reported on exacerbations of low back pain in their PCRT group but did not state any specific numbers or which participants in any group experienced low back pain.

Level of evidence was not established for adverse events due to the data collection variances and analytical differences for each respective study.

### Publication Bias

**Fig. 6:**
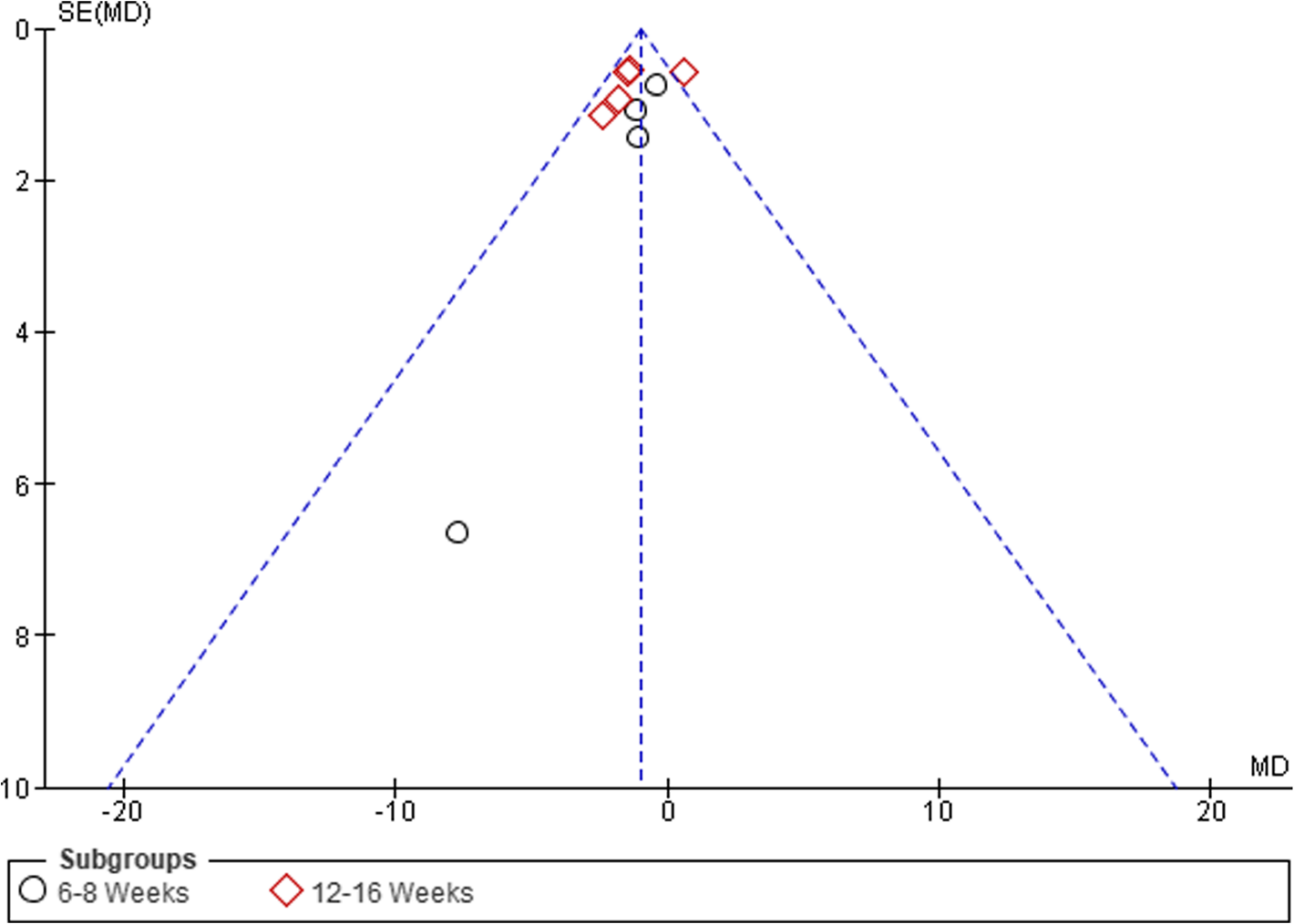
Funnel plot of comparison between GE vs PCRT articles for changes in pain. SE, standard error; MD, mean difference

**Fig. 7:**
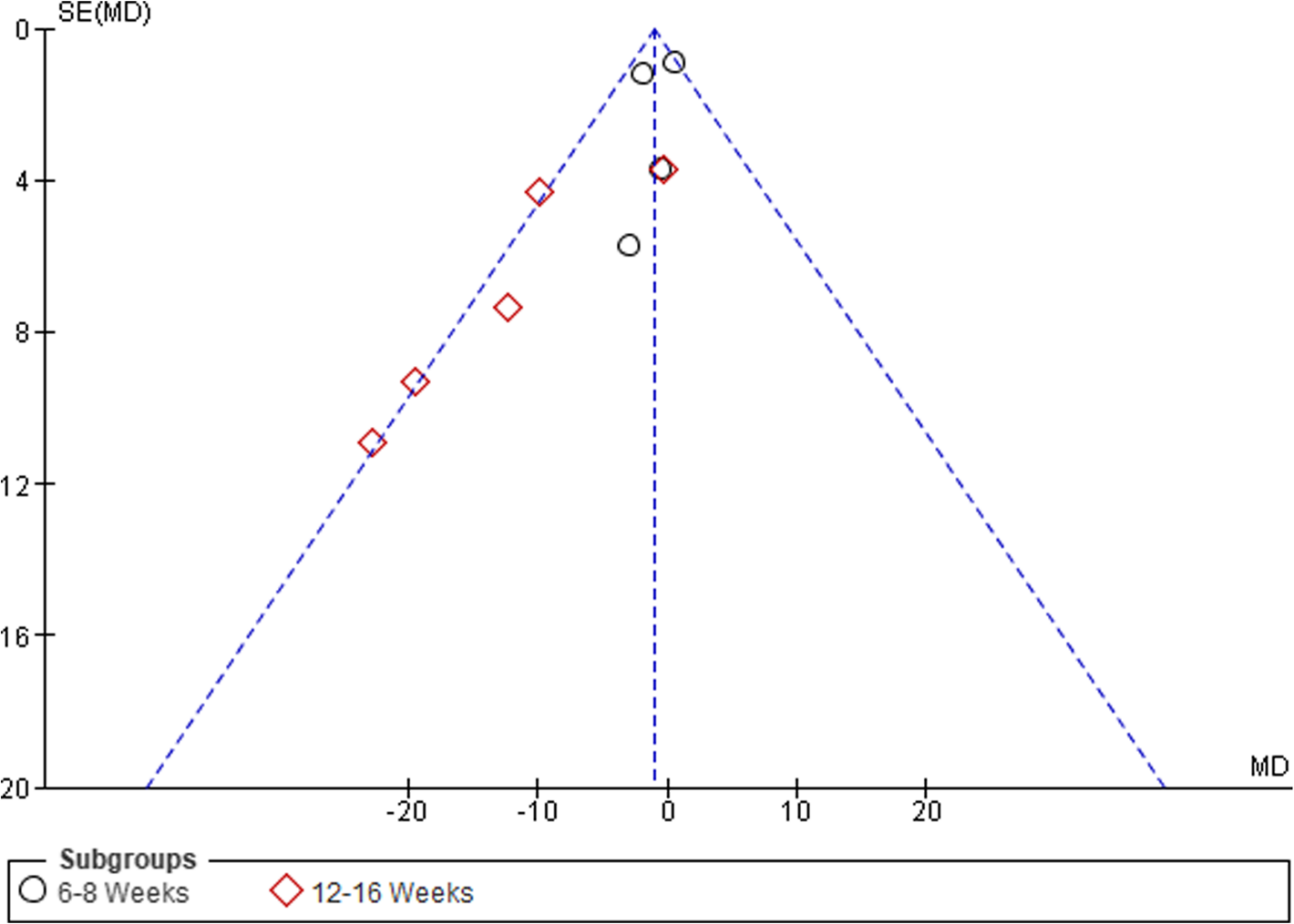
Funnel plot of comparison between GE vs PCRT articles for changes in level of disability. SE, standard error; MD, mean difference

**Fig. 8:**
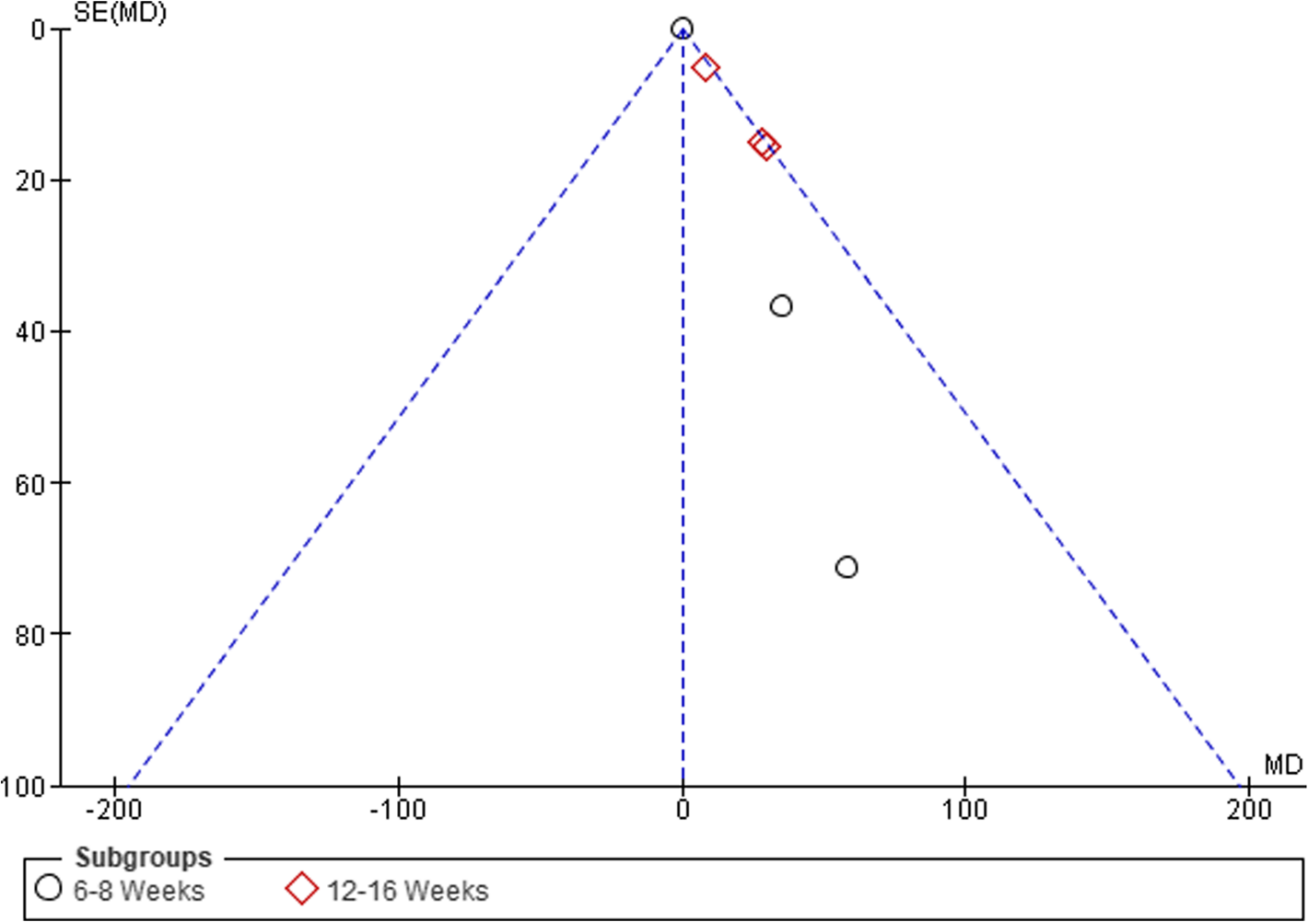
Funnel plot of comparison between GE vs PCRT articles for changes in strength. SE, standard error; MD, mean difference

**Fig. 9:**
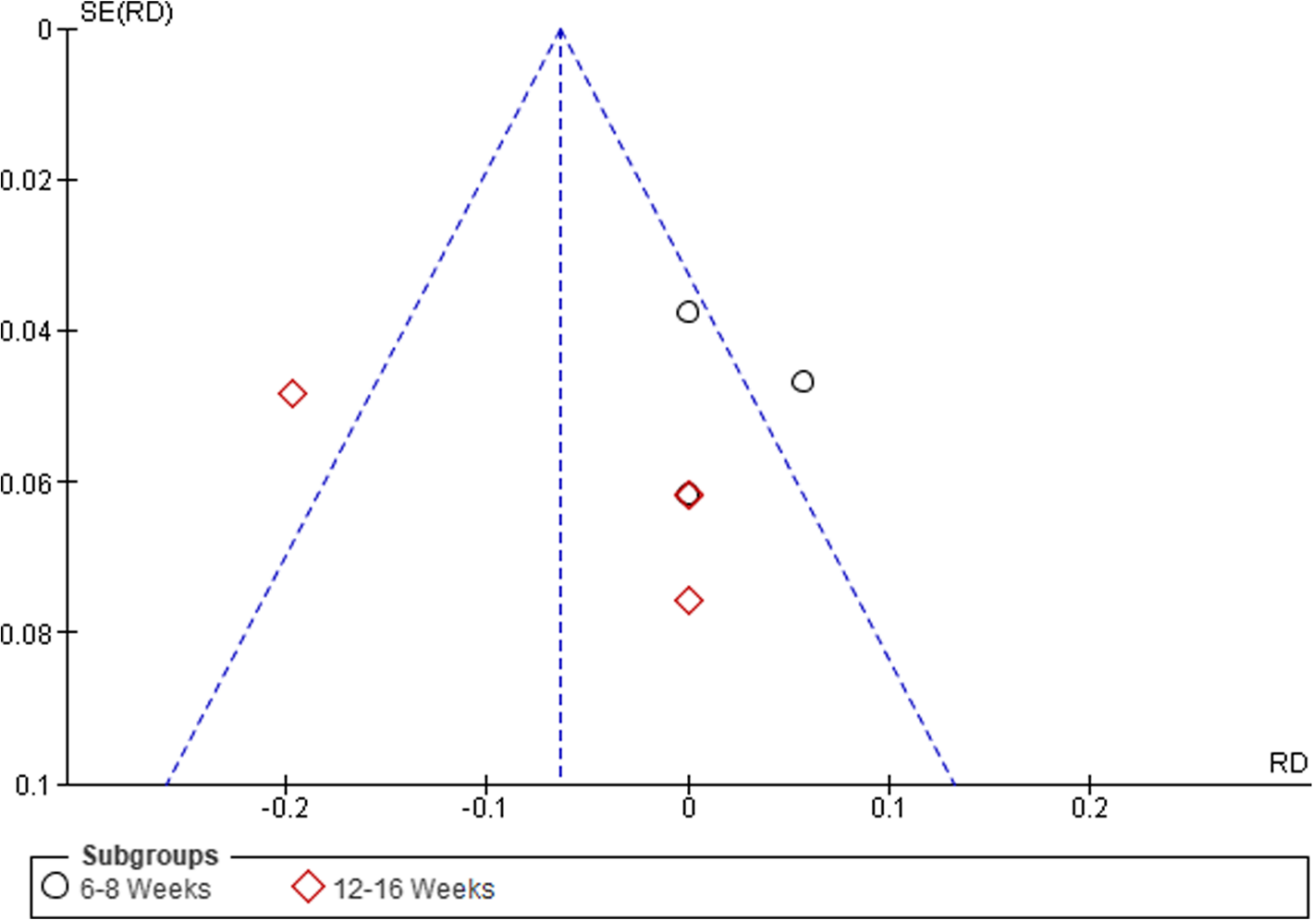
Funnel plot of comparison between GE vs PCRT articles for number of adverse events. SE, standard error; RD, risk difference

Visual inspection of funnel plots was conducted to assess the risk of publication bias, which was considered unlikely (see Figs. 6, 7, 8, and 9).

## Discussion

The primary aim of this review was to determine whether there was any benefit to treating CLBP with PCRT over GE. To the authors’ knowledge, this systematic review and meta-analysis is the first study of this type to have directly compared these two types of exercise interventions in treating this population. This was considered a very important question as while current guidelines state that a variety of exercise forms provide benefit in treating this population, there is currently little evidence indicating the relative efficacy of one type of exercise over another.

This review highlights that there was no difference in the number of adverse events between PCRT and GE programmes and that all the outcomes (pain, disability, muscular strength) improved in patients with CLBP to a significantly greater extent when treated with PCRT over GE, especially when such effects were examined over 12 to 16 weeks. The results of this study show highly favourable outcomes for PCRT relative to GE, especially when assessed over 12 to 16 weeks, a result that might have substantial clinical significance to allied health professionals who work with individuals with CLBP.

It is worth noting that definitions for what “conditions” do and do not fall under the umbrella term of CLBP is a contentious subject that is continually evolving. The definition and exclusion criteria we used were consistent with a majority of the studies we included and previous systematic reviews in this field. Common phrases such as “spinal abnormalities/structural deformities” [14] and “disc protrusions/herniations” [8, 30, 31, 33, 34] were used to exclude potential participants in the trials/study.

### Pain

Participants in the PCRT and GE groups both reported significant reductions in their level of pain. Such results were consistent with previous reviews involving a variety of forms of exercise training programmes in patients with CLBP [8, 42, 43]. Of greater interest, the current study demonstrated that PCRT resulted in significantly greater changes in the level of pain compared to GE.

Using the Furlan et al. [22] levels of evidence approach, there was a strong level of evidence that PCRT produced a larger (small effect) reduction in pain than GE. However, as the length of the exercise interventions increased, the relative difference in the level of pain between PCRT and GE programmes became greater. Specifically, for interventions performed for 12–16 weeks, there was a strong level of evidence that PCRT produced a larger (moderate effect) reduction in pain than GE, whereas only a small effect difference was observed for studies between 6 and 8 weeks in duration. The time dependency of this result may reflect the natural time course of adaptation, whereby injured tissue requires a certain period of time to heal, requiring progressively increased loading to help re-adapt the affected area, encourage growth and stimulate restorative properties in the area to encourage healing [44]. Due to the length of time many of these individuals have lived with a diagnosis of CLBP, it is likely that these individuals had experienced numerous negative morphological and behavioural alterations associated with this condition. Such results may also reflect the potentially greater time required for the patients performing PCRT compared to GE options like walking to develop the appropriate movement competency to sufficiently load their posterior chain muscles in a safe and effective manner.

While most of the individual studies tended to show greater reductions in pain for PCRT compared to GE interventions, Hurley et al. [32] reported results that showed a tendency for greater benefits from GE than PCRT. Unlike the other included studies, this one was the only multisite trial, with the trial carried out across 5 different facilities simultaneously with different researchers/physiotherapists supervising each class. Therefore, in an attempt to standardize the PCRT exercise prescription across the different sites, the actual exercise prescription and the way it was delivered may have been simplified compared to other studies involving PCRT interventions. Specifically, only 2 out of 10 of exercises used added resistance as a final progression, suggesting that the overall intensity and level of progressive overload for the PCRT programme was substantially less than the other PCRT studies included in the current review [32]. Hurley et al. [32] also differed to the other trials by having a higher relative age for the PCRT (mean 45.4, SD = 11.4 years) than GE (mean 34.2, SD = 8.9 years) programme. It is worth noting that removal of Hurley et al. [32] resulted in substantial changes in heterogeneity across all outcomes (*I*^2^ = 0–12%) as well as greater statistical significance in favour of PCRT. While based on only the exclusion of one study, this may suggest that a key feature underlying the potential benefits of PCRT over GE in reducing back pain in patients with CLBP is the use of progressive overload within the PCRT intervention.

### Disability

Previous systematic reviews [8, 42, 43, 45] have demonstrated that similar forms of exercise therapy were able to significantly decrease disability in CLBP populations. A novel finding of the current study was that PCRT demonstrated significantly greater changes in the level of disability than what resulted from GE.

Using the Furlan et al. [22] levels of evidence approach, there was a strong level of evidence that PCRT produced a larger (small effect) reduction in disability than GE. Similar to the results for pain, the relative difference in the level of disability between the PCRT and GE programmes became greater with longer duration programmes. Specifically, for interventions lasting 12–16 weeks, there was strong level of evidence that PCRT produced a larger (moderate effect) reduction in disability than GE, whereas only a small effect difference was observed after 6–8 weeks.

The rationale for the greater benefit of PCRT compared to GE for improving levels of disability in patients with CLBP would reflect similar mechanisms to those underlying the larger improvements in pain seen with PCRT over 12 to 16 weeks. Further, there could be a movement confidence and/or movement competency component that may also be important, whereby the CLBP patient may require a substantially greater period of time to improve their perceptions of disability as such tasks may have previously invoked pain or feelings of inadequacy when performed in public [46].

### Strength

As with pain and disability, the level of muscular strength tended to improve to a greater extent from performing PCRT compared to GE. Previous reviews have shown that increases in muscle strength are possible in this population and may correlate with decreases in pain and level of disability, with exercise specification still unclear [47]. This study demonstrated that PCRT showed significantly greater changes in muscle strength than GE.

Using the Furlan et al. [22] levels of evidence approach, there was a strong level of evidence that PCRT produced a larger (small effect) increase in muscular strength than GE. Like the results for pain and disability, the relative difference in the muscular strength improvements between the PCRT and GE programmes became more pronounced with time. Specifically, for interventions lasting 12–16 weeks, there was a strong level of evidence that PCRT produced a larger (moderate effect) increase in muscular strength than GE, whereas only a small effect difference was observed after 6–8 weeks. This duration-related difference appears consistent with the positive relationship between training time and muscular strength adaptations in CLBP populations identified in a previous systematic review [8].

In order to improve muscular hypertrophy, strength and endurance, we know that muscle and associated connective tissues need to be progressively overloaded in order to adapt. Conversely, when tissues are not stimulated or utilized, the opposite can occur, with muscle wasting and a reduction in muscle strength/endurance [44]. A review by Dreisinger [48] concluded that resistance training was the only exercise intervention that significantly increases muscular strength, flexibility, endurance and balance in patients with CLBP. It is also well understood that resistance training causes greater muscle damage and therefore requires more recovery time than most other forms of exercise. We also know that during the first 2–8 weeks of resistance training in an untrained individual, a majority of the strength gains will be representative of neural adaptations as morphological changes are typically only observed after 8 weeks of training [49–51].

It is worth noting that two studies [33, 34] included in the meta-analysis that utilized correlational analyses reported significant relationships between bench press strength gains and a decrease in pain and level of disability in patients with CLBP. Specifically, Jackson et al. [33] showed that ~ 64% and 59% of the common variance in the decrease in pain and disability, respectively, could be explained by increases in upper body strength. Consistent with the findings for other musculoskeletal injuries such as hamstring or rotator cuff strain injuries, whereby stronger individuals are much less likely to experience such injuries [52, 53], such evidence suggests that clinicians should ensure that their rehabilitation programmes for individuals with CLBP involve a progressive resistance training component to maximize their rehabilitation.

### Adverse Events

There was no statistical difference in the number of adverse events between PCRT (*n* = 2) and GE (*n* = 14) groups. It must however be noted that all these adverse events reported for GE were observed by Hurley et al. [32], with 10 participants reporting back pain, 2 reporting knee pain and 2 reporting ankle pain. Further, 2 of 8 studies did not provide any mention of adverse events, and even though Jackson et al. [33] reported that some adverse events did occur in their study, no numerical data were provided for either exercise group in relation to the number of adverse events recorded.

Regardless of these issues, the lack of any significant difference in the number of adverse events between the PCRT and GE groups may be surprising to many people based on the widely held view that strength training is an activity that is likely to injure the lower back, whereas walking is comparatively low-risk. A recent systematic review by Keogh and Winwood [54] involving athletes competing in strength sports such as weightlifting, powerlifting and bodybuilding demonstrated that while lower back injuries were one of the most commonly injured body sites in these sports, these athletes only averaged ~ 2–4 injuries per every 1000 training hours. Such rates of injury per 1000 h of training are substantially less than reported in many ball sports, further supporting the potential safety of PCRT-focused rehabilitation for individuals with CLBP.

## Study Limitations and Strengths

One potential limitation of this study is the variations in exercise interventions for both PCRT and GE. Thus, what constitutes the optimal form of PCRT in terms of exercise selection, loads, sets, repetitions and rest periods is still relatively unknown. The PCRT programmes that were included in this meta-analysis were typically performed 1–3 times per week, with each exercise performed for 2–3 sets of 8–12 repetitions. The type of load used in these exercise prescriptions also varied, with different interventions using free weights, machines and bodyweight, and some using a combination of equipment and bodyweight. While most studies included lower limb and hip strengthening exercises, this was not uniform across all studies. This could also be interpreted as a strength of this meta-analysis, as it shows that a variety of PCRT exercises and protocols can be utilized with similar effects.

Another limitation of this study is the variance in the GE comparator groups. With 3 groups utilizing other forms of resistance training [30, 31, 36], 4 using AT [32–35], and one using ADLs [14], it leaves the control groups open to variance and makes it difficult to distinguish if either one of these GE interventions is less effective than the other. This could possibly be leading to an unintentional bias towards PCRT if one of these comparators is less effective than the others. This also makes it difficult to distinguish which of the PCRT protocols is more effective than the others due to the variance in the comparator protocols. However, previous RCTs involving different active therapies have suggested that specificity of training may not be the primary mechanism underlying improvement in this population; rather a “central” effect involving changing perceptions with their level of pain and disability may be more important [10].

Another limitation of this study was the estimation of some data points that were not published in the original articles. As stated earlier, the *p* values were set at less than 0.05 for “significant effects” and less than 0.1 for “non-significant effects” when not explicitly stated in the articles. While set at very conservative values to try and dissuade as much influence from the authors as possible, this leads to probable underestimation of effect sizes for each of the outcomes in favour of PCRT where this was utilized. This in turn leads to an effect size and magnitude that is probably diminished due to the use of these conservative values and SMD.

The final limitation of the manuscript may concern the subgroup analyses and the relative heterogeneity of some of these results. When additional studies in this area are conducted and incorporated into updated meta-analyses, the larger sample sizes may increase the confidence in the subgroup analyses and their clinical implications.

Due to the recognition of these limitations, several recommendations for future research have emerged as a result. These can hopefully guide future RCTs and clinical practice guidelines in this area of study and practice. Given the variance in exercise interventions, a standardized exercise protocol looking at only using PCRT exercises with no other exercises or interventions should be utilized and compared against walking programmes, mixed resistance training and a usual care control group. This would greatly aid in guiding effective treatment for the CLBP population. The significant correlation between increases in muscular strength with decreases in pain and level of disability in patients with CLBP should also be explored going forward, so to determine if the magnitude of the improvements in pain and disability are reflective of increases in strength. Another study aim should be to clearly distinguish patient activity levels prior to joining the study to aid future research in answering how much of an effect prior training/activity levels influence outcomes in exercise-based interventions for CLBP.

## Conclusion

The results of this study show that treating patients suffering with CLBP within the recreationally active/sedentary population using PCRT is significantly more effective than using GE. The results of this study show there are overall “strong” levels of evidence for significant improvements in pain, level of disability and strength with PCRT. These results also showed no significant greater risk of adverse events, relative to general exercise or walking programmes, although this is based on a lower number of studies that adequately reported definitions, data collection methods and group-based results for adverse events, leading to possibly substantial heterogeneity in this outcome. The evidence within the current body of literature favours using PCRT over a 12–16-week period as opposed to 6–8 weeks, with significantly greater improvements in pain, level of disability and strength observed with additional training. Future research should be aimed at high-quality RCTs, isolating exercise interventions based specifically around PCRT only, and comparing these against a control group, aerobic exercise interventions such as walking, and/or a mixed-GE intervention. Researchers should also account for prior activity levels in their patients in order to better aid clinicians in treatment direction, based on efficacy of interventions with populations of varying activity levels.

## Data Availability

All data generated or analysed during this study are included in this published article [and its supplementary information files].

## Abbreviations

CLBP: Chronic low back pain
PCRT: Posterior chain resistance training
GE: General exercise
JBI: Joanna Briggs Institute
RCTs: Randomized controlled trials
SMD: Standardized mean difference
CI: Confidence interval
RD: Risk difference
PRISMA: Preferred Reporting Items of Systematic Reviews and Meta-Analyses
SD: Standard deviation
ADL: Activities of daily living
RM: Repetitions max
